# Maximum Expected Information Approach for Improving Efficiency of Categorical Loudness Scaling

**DOI:** 10.3389/fpsyg.2020.578352

**Published:** 2020-11-17

**Authors:** Sara E. Fultz, Stephen T. Neely, Judy G. Kopun, Daniel M. Rasetshwane

**Affiliations:** Center for Hearing Research, Boys Town National Research Hospital, Omaha, NE, United States

**Keywords:** loudness, loudness perception, psychoacoustics, maximum likelihood, categorical loudness scaling

## Abstract

Categorical loudness scaling (CLS) measures provide useful information about an individual’s loudness perception across the dynamic range of hearing. A probability model of CLS categories has previously been described as a multi-category psychometric function (MCPF). In the study, a representative “catalog” of potential listener MCPFs was used in conjunction with maximum-likelihood estimation to derive CLS functions for participants with normal hearing and with hearing loss. The approach of estimating MCPFs for each listener has the potential to improve the accuracy of the CLS measurements, particularly when a relatively low number of data points are available. The present study extends the MCPF approach by using Bayesian inference to select stimulus parameters that are predicted to yield maximum expected information (MEI) during data collection. The accuracy and reliability of the MCPF-MEI approach were compared to the standardized CLS measurement procedure (ISO 16832:2006, 2006). A non-adaptive, fixed-level, paradigm served as a “gold-standard” for this comparison. The test time required to obtain measurements in the standard procedure is a major barrier to its clinical uptake. Test time was reduced from approximately 15 min to approximately 3 min with the MEI-adaptive procedure. Results indicated that the test–retest reliability and accuracy of the MCPF-MEI adaptive procedures were similar to the standardized CLS procedure. Computer simulations suggest that the reliability and accuracy of the MEI procedure were limited by intrinsic uncertainty of the listeners represented in the MCPF catalog. In other words, the MCPF provided insufficient predictive power to significantly improve adaptive-tracking efficiency under practical conditions. Concurrent optimization of both the MCPF catalog and the MEI-adaptive procedure have the potential to produce better results. Regardless of the adaptive-tracking method used in the CLS procedure, the MCPF catalog remains clinically useful for enabling maximum-likelihood determination of loudness categories.

## Introduction

Loudness is the perceptual correlate of the physical intensity of a sound (Fletcher and Munson, 1933). A variety of psychometric procedures may be used to quantify loudness in humans, including but not limited to: loudness matching, magnitude estimation, cross-modality matching, and loudness scaling (Cox, 1989; Kollmeier and Hohmann, 1995). Categorical loudness scaling (CLS) is a procedure in which listeners assign meaningful labels to stimuli of varying intensities as a means of estimating loudness growth with increasing stimulus level (Brand and Hohmann, 2002; ISO 16832:2006, 2006). Measurements of loudness perception offer insight into auditory health because they become altered when the cochlea is damaged (e.g., Allen, 2008). CLS has often been used for studying loudness perception in listeners with sensorineural hearing loss due to both its ease of testing and validity (Al-Salim et al., 2010; Rasetshwane et al., 2015, 2018; Oetting et al., 2016). CLS measurements have been used to assess loudness perception in patients with tinnitus (Hébert et al., 2013) and hyperacusis, which is a reduced tolerance to loud sounds (Noreña and Chery-Croze, 2007). CLS procedures have also been used to evaluate abnormalities in loudness perception in patients with autism (Khalfa et al., 2004) and in concussed athletes (Assi et al., 2018).

New hearing aid users often have complaints about the loudness and annoyance of certain sounds. Although abnormal loudness perception is a driving factor in dissatisfaction with hearing aids (Blamey and Martin, 2009), loudness is not typically measured during the clinical hearing aid fitting process. This is in part due to concerns related to the reliability, accuracy, and test time required to obtain loudness measures, and because the nature of suprathreshold variability across listeners is not yet fully understood (Elberling, 1999; Al-Salim et al., 2010).

Several procedures have been used in previous studies to calculate a CLS function from trial-by-trial data. These include (1) fitting a loudness model (two segment straight lines) to the trial-by-trial data (e.g., Brand and Hohmann, 2002; Heeren et al., 2013; Oetting et al., 2014), and (2) fitting a model to the median of the trial-by-trial data (Al-Salim et al., 2010; Rasetshwane et al., 2015). It has been noted that these procedures can lead to over-smoothing of the data (Trevino et al., 2016a; Wròblewski et al., 2017) and that using the median of trial-by-trial data may produce more reliable results. In the current study, we follow the method described in Trevino et al. (2016a).

We previously developed a probability model of CLS that characterizes loudness-category selection as a multi-category psychometric function (MCPF) (Trevino et al., 2016a), which is a generalization of the commonly used two-category psychometric function. The MCPF provides a more comprehensive characterization of the variability associated with listener responses because it combines all categories into a single framework. The MCPF provides a statistical basis for smoothing listener responses across categories that supports a maximum-likelihood determination of loudness-category boundaries for a given set of responses. The MCPF adds a new dimension to CLS data and facilitates parameterization of suprathreshold variability across listeners. In the present study, we extend the MCPF approach by using Bayesian inference to select stimulus parameters that are predicted to yield maximum expected information (MEI) during data collection.

We then assess the test–retest reliability and accuracy of an adaptive procedure that utilizes a limited number of trials for MCPF-MEI. Test–retest reliability was assessed across two visits. For assessment of accuracy, the International Standards Organization (ISO) fixed-level procedure, which utilizes numerous trials, served as the reference procedure for estimating a listener’s CLS function (Brand and Hohmann, 2002; Kinkel, 2007). Improving the reliability and accuracy of CLS procedures may enhance the clinical acceptability of loudness measurements and potentially improve hearing aid fitting methods.

Entropy is an information-theoretic concept that quantifies the randomness (or uncertainty) of a system that has many possible states. The entropy of any system has its maximum value when all possible states are equally likely. Entropy is reduced when information becomes available that makes some states more likely than other states. Thus, entropy and information have a complementary relationship. Information increase is always associated with an equal amount of entropy reduction. In the context of CLS measurements, each trial, which consists of a listener’s response to a particular stimulus, provides a small amount of new information about the listener’s loudness perception. When listener responses are reliable (e.g., when listener responses are monotonic functions of stimulus level), the accumulated information increases, and the entropy is reduced, as the number of trials increases. This study investigated the idea that the efficiency of a CLS test could be improved by selecting the stimulus for each trial that is expected to provide the maximum amount of information from the response portion of that trial.

In this study, we compared two different adaptive-tracking methods: (1) the standard CLS method described by ISO 16832:2006 (2006) and (2) the MEI method. The “gold-standard” for this comparison was a non-adaptive, fixed-level method, which was not considered to be clinically viable because it required too much time. A further comparison was included in the method used to construct the MEI loudness functions from the trial-by-trial data: (1) median sound pressure level (SPL) within each loudness category and (2) maximum likelihood (ML) MCPF.

## Materials and Methods

### Participants

Forty-five adults participated in this study (23 female). The demographic makeup of our sample was 91.9% Not Hispanic, 4.4% Hispanic, and 4.4% Not Reported. The participants were 77.8% White, 11.1% Black, 0% American Indian and Alaska Native, 0% Asian, 0% Native Hawaiian and Other Pacific Islander, 4.4% Two or More Races, and 6.7% Not Reported. According to the United States Census 2018 American Community Survey, the demographic makeup of our local community, Omaha, NE is 85.3% Not Hispanic and 14.7% Hispanic. The city population is 77.0% White, 12.1% Black, 0.9% American Indian and Alaska Native, 3.7% Asian, 0.0% Native Hawaiian and Other Pacific Islander, and 3.6% Two or More Races. The demographic makeup of the United States is 81.5% Not Hispanic and 18.5% Hispanic. The population is 72.2% White, 12.7% Black, 0.9% American Indian and Alaska Native, 5.6% Asian, 0.2% Native Hawaiian and Other Pacific Islander, and 3.4% Two or More Races (American Community Survey 2018). All participants reported English as their primary language.

All participants were recruited from a database of potential research participants that is maintained by Boys Town National Research Hospital (BTNRH). Data collection was conducted under a protocol that was approved by the BTNRH Institutional Review Board. Informed consent was obtained prior to testing and participants were compensated for their participation.

**FIGURE 1:**
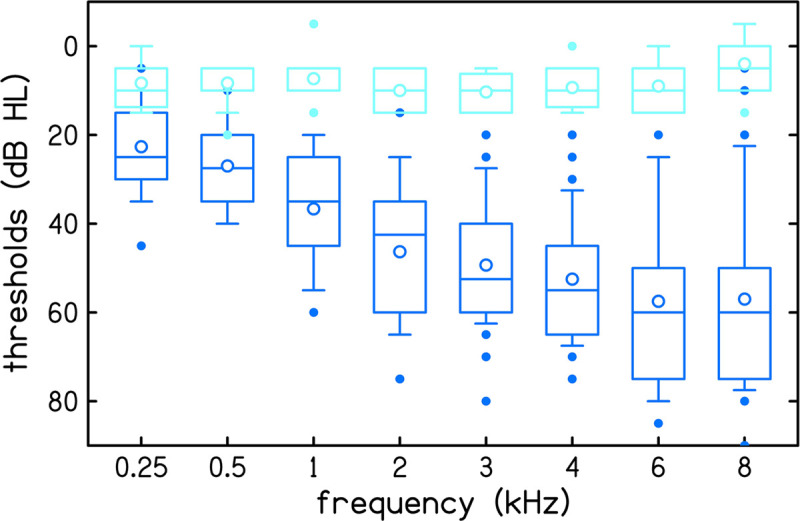
Audiometric thresholds of 15 normal hearing participants (light blue) and 30 participants with hearing loss (dark blue). Boxes represent the interquartile range and whiskers represent the 10th and 90th percentiles. Outliers, defined as data points that are outside the 10th to 90th percentile range, are plotted using filled circles. Within each box, lines represent the median and open circles represent the mean.

Audiometric thresholds were measured at eight frequencies (0.25, 0.5, 1, 2, 3, 4, 6, and 8 kHz) with an audiometer (GSI AudioStar Pro, Grason-Stadler, Eden Prairie, MN, United States) using ER3A headphones (Etymotic Research, Elk Grove Village, IL, United States) following the Hughson-Westlake procedure (American Speech-Language-Hearing Association [ASHA], 1978). Participants were classified as having normal hearing when thresholds in the test ear were ≤15 dB HL at all audiometric frequencies. Participants were classified as having sensorineural hearing loss when thresholds in the test ear were ≥20 dB HL at both of the test frequencies used for the CLS procedures, 1 and 4 kHz. Fifteen participants had normal hearing (age range 21–74, mean 43 years) and thirty participants had hearing loss (age range 23–74, mean 55 years). Participants with sensorineural hearing loss had audiometric thresholds ≤75 dB HL at the test frequencies for the CLS procedures. The distribution of audiometric thresholds is displayed in Figure 1.

All participants had normal middle-ear status in the test ear based on normal otoscopic inspection, normal 226-Hz tympanogram, and air-bone gaps ≤10 dB from 0.5 to 4 kHz. The inclusion criteria for tympanometry (Madsen Otoflex 100, GN Otometrics, Denmark) required peak-compensated static acoustic admittance between 0.3 and 2.5 mmhos and peak tympanometric pressure between −100 and +50 daPa.

All CLS testing was conducted monaurally. If both ears met the inclusion criteria, the better ear was selected for testing. If the thresholds were symmetrical, the test ear was selected randomly, though there was an attempt to balance the number of left and right ears. Overall, data were collected from 21 right and 24 left ears.

### Procedures

Participants were seated in a sound-treated room. Pure-tone stimuli (1 and 4 kHz) at levels ranging from 0 to 110 dB SPL were presented monaurally for each of three CLS procedures: (1) fixed-level procedure, (2) slope-adaptive procedure, and (3) MEI-adaptive procedure. Pure tones were 1000 ms in duration with a 20 ms rise/fall time. Stimuli were generated using custom-designed software (MATLAB) that controlled a 24-bit soundcard (Babyface Pro, RME) and were presented to the participants’ ear with an insert earphone (ER3A; Etymotic Research, Elk Grove, MN).

**FIGURE 2:**
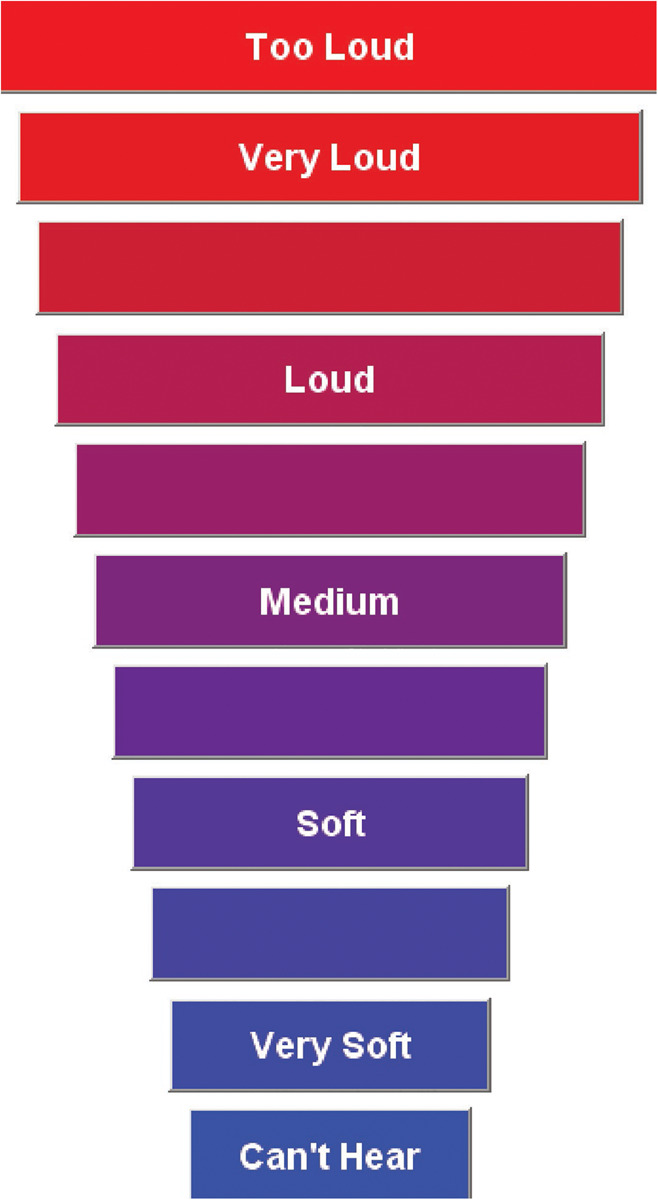
Display of the categorical loudness scale with 11 response categories. This is displayed on a computer monitor used by participants to rate the loudness of the signal. The horizontal bars increase in width from the softest level to the loudest level. This figure appeared previously in Rasetshwane et al. (2015).

The CLS procedure closely followed the ISO standard (ISO 16832:2006, 2006), though it was not our intention to replicate it exactly as described. The procedure determined the level of sounds that corresponded to 11 different loudness categories, with seven of these categories assigned meaningful labels (“Can’t Hear,” “Very Soft,” “Soft,” “Medium,” “Loud,” “Very Loud,” and “Too Loud”). The categories were graphically displayed on a computer monitor as colored horizontal bars that increased in length from bottom (“Can’t Hear”) to top (“Too Loud”). The response window is displayed in Figure 2. After listening to each stimulus, participants selected a category that best represented their perception of the loudness of the sound. Participants were instructed to select “Too Loud” if the sound was loud enough that they wouldn’t want to hear it again and “Very Soft” if the sound was just detectable. The labels used for boundary categories are different than the labels used in the ISO 16832:2006 (2006) but matched those used in our previous studies (Rasetshwane et al., 2015, 2018). However, it should be noted that the ISO standard is open to the use of different labels, including symbols. For the purpose of numerical representation, the 11 loudness categories were assigned categorical units (CUs) ranging from 0 (“Can’t Hear”) to 50 (“Too Loud”) in steps of 5. This numerical representation was not shown to participants.

Participants completed one practice run at one frequency, 1.5 kHz, of either the slope-adaptive or MEI-adaptive procedure, selected randomly. Six conditions were then collected (1 and 4 kHz for each of the three procedures), with the procedure and test frequency randomized for each participant. Data collection was repeated over two visits separated by at least 1 day and up to 42 days. The average number of days between visits was 10.

The CLS test included two stages. The participant’s dynamic range was determined in the first stage, in the first stage, and a loudness function was measured in the second stage. The procedure for determining the dynamic range was the same for all three CLS methods. In this procedure, two sequences of stimuli were interleaved, one sequence ascending in level and the other descending in level. The lower end of a participant’s dynamic range was based on the last audible level (“Very Soft” category) of the descending sequence, while the upper end was based on the last level of the ascending sequence that was not judged as “Too Loud.” The starting level was set equal to the midpoint of the participant’s dynamic range.

Procedures for measuring the loudness function differed by CLS method. For the non-adaptive, fixed-level procedure, up to 22 distinct levels spanning the dynamic range were presented in 5 dB steps. The exact number of levels depended on the listener’s dynamic range. Each level was repeated 10 times, for a total of up to 220 trials. Levels were randomized with restrictions that the same level was never presented consecutively and differences between consecutive levels never exceeded 45 dB.

For the adaptive procedures (slope-adaptive and MEI-adaptive), nine levels within the dynamic range were presented. The run of nine trials was repeated five times, for a total of 45 trials. In the slope-adaptive procedure, the nine levels evenly spanned the dynamic range. In the MEI-adaptive approach, MEI was used to select the next stimulus level as the one that minimized entropy based on the MCPF catalog. In contrast to the fixed-level procedure, the dynamic range of the presentation levels was not fixed during the test for the adaptive procedures. The listeners were instructed to select “Too Loud” if they felt the sound was loud enough that they did not want to hear it again. Thus, whenever a listener responded with “Too Loud,” the upper limit was reduced by 5 dB for the next run to avoid presenting uncomfortable loud sounds. If a listener did not respond with “Too Loud” to any of the nine levels within a run, then the upper limit of the dynamic range was increased by 5 dB for the next run, but never exceeded the 110 dB SPL limit.

A catalog of MCPFs that represent a wide range of potential listeners was created based on fixed-level trial-by-trial data obtained at two frequencies (1 and 4 kHz) from 16 listeners with normal hearing and 25 listeners with sensorineural hearing loss (Trevino et al., 2016a). MCPF generalizes the concept of a psychometric function (the probability of a particular response in a two-alternative paradigm as a function of an experimental variable) to more than two possible responses and represents the probability distribution across multiple response categories as a function of an experimental variable (e.g., Torgerson, 1958). Within the context of CLS data, a MCPF described how loudness category probabilities change with stimulus level. The Trevino et al. catalog has a total of 1460 MCPFs entries. The MEI-adaptive procedure used MEI to select the next stimulus level as the one that minimized entropy based on the MCPF catalog.

Entropy is an information-theoretic measure of how much information is needed to determine an unknown variable (i.e., the *uncertainty* of the variable). In this case, the listener’s CLS function is the unknown variable. At the beginning of the experiment, no prior information is known, many CLS functions are equally probable, and thus the entropy is at a maximum. With each stimulus-response trial, some CLS functions can be determined to be more statistically probable than others, and the entropy is reduced. For each additional trial, the stimulus level that leads to the most entropy reduction is the one that provides the maximum information. MEI is an iterative algorithm that uses the catalog of parameterized CLS psychometric functions to calculate entropy. With each stimulus-response trial, the probability of each potential CLS psychometric function is updated. After updating, the probability-weighted expected entropy of all experimental stimulus levels is computed. The stimulus level with the greatest expected entropy reduction (i.e., provides the MEI) is selected as the level for the following stimulus presentation.

The calculation of entropy is based on posterior probability distribution. At the start of each track, prior to the first trial, each catalog entry is assumed to be equally likely. With each stimulus-response trial, the probability of each potential CLS psychometric function is updated which alters the distribution of probabilities associated with MCPF catalog entries. The procedure for updating the likelihood of each entry after each trial was described by Trevino et al. (2016a). A probability for each entry was calculated by dividing the likelihood for each entry by the sum of the likelihoods for all entries. Prior probabilities are transformed into posterior probabilities by applying relevant conditional probabilities contained in the catalog. The entropy of each posterior probability distribution was calculated by the usual definition as minus the expected value of the log (base 2) of the entry probability. Thus, this entropy decreases with each additional stimulus-response trial.

### Analyses

Loudness-growth functions (loudness in CU as a function of SPL) were generated for each participant from their trial-by-trial responses. The term *trial* refers to a single stimulus/response pair. For all three procedures, CLS functions were obtained by calculating the median SPL for each CU. Unlike our previous procedures (Al-Salim et al., 2010; Rasetshwane et al., 2015), outliers were not removed. However, the drawback is the median value may be based on a single response for sparse data. In addition to calculating a loudness function based on the median SPL for each CU, ML estimation was used to select one MCPF from the catalog that was the best fit to each listener’s responses. Each MCPF describes all boundaries between adjacent loudness categories as individual psychometric functions. The 50% on each of these boundary functions was used to construct conventional CLS loudness growth functions. Although the MEI-ML procedure was intended to be an update of the MEI-Med method, it was not known prior to the study how the two methods would compare, therefore, both methods were applied to the data. Thus, there were a total of four CLS functions: fixed-level, slope-adaptive, MEI-Median (Med) and MEI-ML. See Trevino et al. (2016a) for detailed descriptions of the MCPF procedure and its development.

For analysis purposes, CUs were converted to phons based on the conversion function of Rasetshwane et al. (2015). Besides being the international standard unit for loudness level, phon has the advantage (over CUs) of being a continuous function of stimulus level, which is desirable when computing slopes (ISO 226:2003, 2003). Data for 0 and 50 CUs were not included in analysis because the levels corresponding to these loudness categories are unbounded. For example, if a listener judged 100 dB SPL as “Too Loud” (50 CU), then we would expect that listener to also judge all levels >100 dB SPL as “Too Loud.”

Estimates of hearing threshold were derived from the CLS functions as the stimulus level corresponding to 2.5 CUs through simple linear regression using data for CU ≤ 20. This portion of the loudness function varies linearly with level, as was previously demonstrated (Al-Salim et al., 2010; Oetting et al., 2014). Because 2.5 CU is midway between 0 CU (“Can’t Hear”) and 5 CU (“Very Soft”), the estimate of threshold is equivalent to a condition in which the stimulus was audible 50% of the time. This definition of threshold is consistent with that used by Trevino et al. (2016a), in which threshold was defined as the inflection point between 0 and 5 CU. There were instances for the MEI-Med procedure when the CLS function did not have any data for CU ≤ 20. When this occurred, the lowest level that the participant responded was used as the estimate of CLS threshold. This occurred for two participants at 1 kHz and three participants at 4 kHz.

Audiometric thresholds, obtained in dB HL, were converted to dB SPL for analysis based on reference level equivalents for insert earphones (American National Standards Institute, 2010).

Reliability was assessed by comparing CLS functions between the first visit and second visit for each of the four procedures. Accuracy was assessed by comparing CLS functions for the adaptive procedures to CLS functions for the fixed-level procedure including data from both visits. The fixed-level procedure was the reference for accuracy assessment because it had a larger number of trials compared to the adaptive procedures. Both reliability and accuracy were quantified using a comprehensive set of statistical methods including (1) Bland-Altman bias, (2) Cronbach’s α, and (3) root mean square error (rmse).

Bland-Altman plots show the distribution of differences between two sets of measurements. The bias represents systematic error and should be close to 0 for repeatable measurements. The plots also show 95% limits of agreement (LOA) between measurements, calculated as mean ±1.96 standard deviation (SD) when the differences are uniformly distributed and as mean ±2 SD when the differences are not uniformly distributed (Bland and Altman, 1986, 1999). The Kolmogorov-Smirnov test (Massey, 1951) indicated that differences for all conditions were normally distributed. Thus, the 95% LOA were calculated as mean ±1.96 SD. A 95% confidence interval of bias that does not include the line of equality (zero line) indicates a significant systematic error. It is worth noting that, although the Bland-Altman method is a useful tool for assessing similarities between two data sets, it does not provide criterion for acceptable bias or LOA. Interpretation of the Bland-Altman plots often requires some *a priori* information or assumptions related to the clinical or research question.

Cronbach’s α is a coefficient of reliability that measures how closely a set of measurements are related (Cronbach, 1951). Values of Cronbach’s α can be interpreted as follows: α: ≥ 0.9 = excellent, ≥ 0.8 = good, ≥ 0.7 = acceptable, ≥ 0.6 = questionable, ≥ 0.5 = poor, and <0.5 = unacceptable (George and Mallery, 2003).

Although the participants were encouraged to use all 11 response categories in their loudness judgments, some participants did not use all categories. In those cases, there were missing data for the categories that were not utilized by the listener. Most of these instances occurred for CUs of 40 and 45. Some conditions in the dataset were missing due to tester error in data collection. These included the MEI procedure at 4 kHz for one participant with normal hearing and the slope-adaptive procedure at 4 kHz for two participants with hearing loss. Additionally, one participant with hearing loss did not return for the second visit. These conditions were excluded from analysis. Overall, 3.4 and 4.6% of data were missing for the fixed-level procedure at 1 and 4 kHz, respectively; 5.9 and 8.4% of data were missing for the slope-adaptive procedure at 1 and 4 kHz, respectively; 9.8 and 11.9% of data were missing for the MEI-Med procedure at 1 and 4 kHz, respectively; and 5.6 and 1.1% of data were missing for the MEI-ML procedure.

## Results

The test time for the adaptive procedures was significantly less than required for the fixed-level procedure. The mean test time for the fixed-level procedure was 15 min, 0 s (range 6 min, 3 s to 26 min, 34 s). The mean test time for the slope-adaptive procedure was reduced to 2 min 47 s (range 2 min, 0 s to 4 min, 7 s). The mean test time for the MEI procedure was reduced to 2 min, 35 s (range 1 min, 49 s to 4 min, 33 s).

**FIGURE 3:**
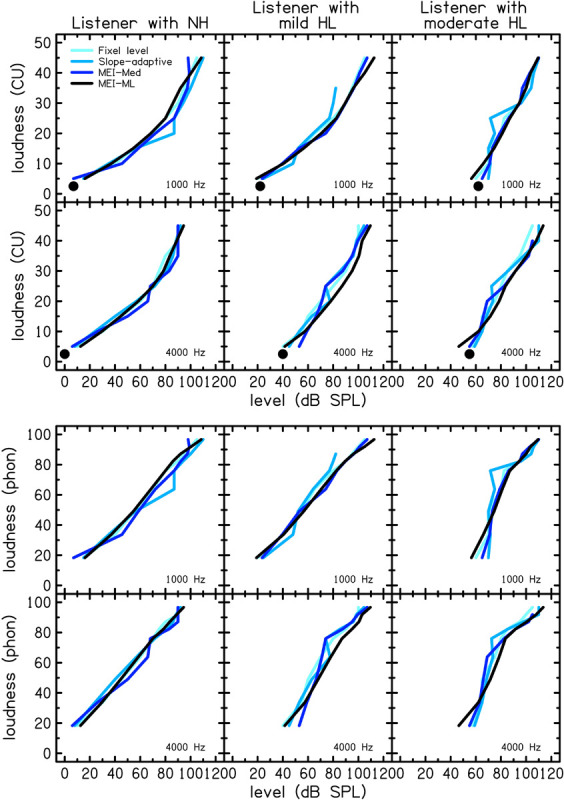
CLS functions for each of the procedures at 1 (top row) and 4 kHz (bottom row) for three individual (representative) participants with normal hearing (NH; left column), mild hearing loss (HL, middle column), and moderate HL (right column). The top set of six panels show loudness in categorical units and the bottom set of six panels show loudness level in phons. The participants’ audiometric thresholds are indicated by a black filled circle.

Figure 3 shows CLS functions for each of the procedures at 1 kHz (top rows) and 4 kHz (bottom rows) for three individual participants: one with normal hearing (NH), one with mild sensorineural hearing loss (HL), and one with moderate sensorineural hearing loss. The functions are created from averages of measurements collected over two visits. The top set of six panels display loudness in CUs and the bottom set of six panels display loudness level in phons. The participants’ audiometric thresholds are indicated by a solid circle. CLS functions are shifted to the right with increasing degrees of hearing loss. The MEI-ML method is thought likely to be more reliable than MEI-Med because its estimates are smoothed across categories.

**FIGURE 4:**
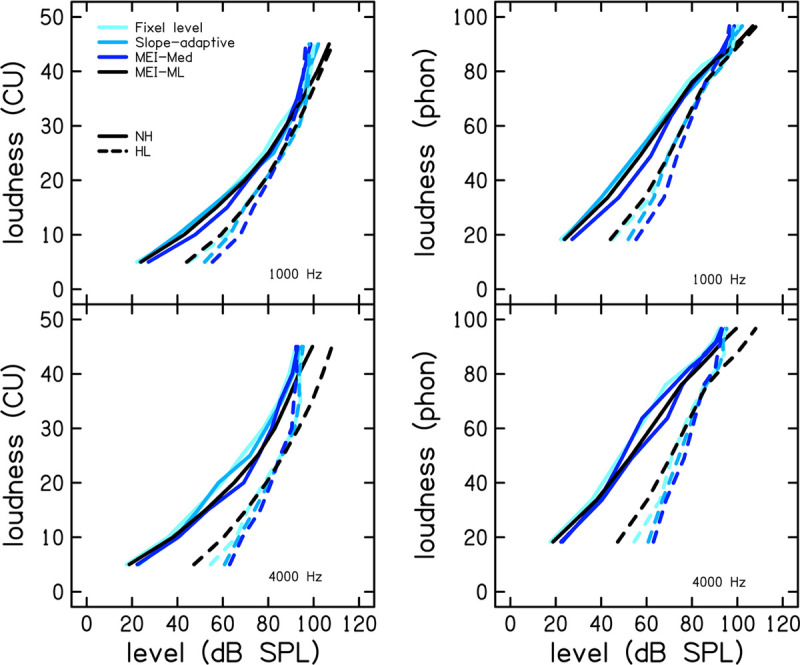
Mean CLS functions for each of the procedures at 1 (top row) and 4 kHz (bottom row) for the group of participants with normal hearing (NH; solid lines) and the group with hearing loss (HL; dashed lines). The left column shows loudness in categorical units and the right column shows loudness level in phons. CLS functions were calculated based on the mean sound pressure level (SPL) per category (CU).

Figure 4 shows mean CLS functions for each of the procedures at 1 (top row) and 4 kHz (bottom row) for the group of participants with normal hearing (NH; solid lines) and the group with hearing loss (HL; dashed lines). The left panels display loudness in CUs and the right panels display loudness level in phons. CLS functions were calculated from the mean of median SPL for each CU. There were similarities between the procedures. On average, participants with hearing loss have a reduced dynamic range compared to participants with normal hearing. CLS functions are shifted to the right for the group of listeners with hearing loss. The variability of the loudness function was assessed using SD, calculated separately for each CU. To avoid clutter, the SDs are presented in Tables 1–4 instead of as error bars in Figure 4. Specifically, Tables 1, 2 show SDs for participants with normal hearing at 1 and 4 kHz, respectively, and Tables 3, 4 show SDs for participants with hearing loss at 1 and 4 kHz, respectively. Values are given in dB. Across procedures, SDs were higher for lower CUs compared to higher CUs. The variability was similar between participants with NH and HL at 1 kHz but was increased for participants with hearing loss at 4 kHz.

**TABLE 1:** Standard deviations of sound pressure level (SPL) for each categorical unit (CU) for participants with normal hearing for each of the four CLS procedures at 1 kHz.

**CU**	**5**	**10**	**15**	**20**	**25**	**30**	**35**	**40**	**45**	**Mean**
Fixed-level	7.79	10.68	10.01	8.54	9.08	11.93	7.35	9.12	6.50	9.00
Slope-adaptive	11.76	11.70	11.90	14.07	10.99	12.17	9.43	8.48	7.55	10.90
MEI-Med	14.52	13.13	9.64	12.78	9.89	8.59	6.28	6.17	4.88	9.54
MEI-ML	12.27	13.42	12.44	10.74	8.87	7.51	6.91	7.12	7.43	9.63
Mean	11.59	12.24	11.00	11.53	9.71	10.05	7.49	7.72	6.59	9.77

*Values are given in dB.*

**TABLE 2:** Standard deviations of sound pressure level (SPL) for each categorical unit (CU) for participants with normal hearing for each of the four CLS procedures at 4 kHz.

**CU**	**5**	**10**	**15**	**20**	**25**	**30**	**35**	**40**	**45**	**Mean**
Fixed-level	8.14	11.39	10.62	11.21	9.78	9.39	8.11	7.94	6.38	9.22
Slope-adaptive	14.08	12.06	13.29	12.27	12.37	11.36	8.73	7.58	6.07	10.87
MEI-Med	11.63	13.52	16.14	10.16	9.40	7.54	6.30	7.55	4.94	9.69
MEI-ML	8.02	12.46	13.10	11.85	10.07	8.38	7.35	6.57	6.13	9.32
Mean	10.47	12.36	13.29	11.37	10.40	9.17	7.62	7.41	5.88	9.77

*Values are given in dB.*

**TABLE 3:** Standard deviations of sound pressure level (SPL) for each categorical unit (CU) for participants with hearing loss for each of the four CLS procedures at 1 kHz.

**CU**	**5**	**10**	**15**	**20**	**25**	**30**	**35**	**40**	**45**	**Mean**
Fixed-level	11.19	9.74	8.70	11.04	10.91	10.42	10.31	9.70	8.53	10.06
Slope-adaptive	13.88	10.76	10.62	11.16	10.59	10.82	10.28	9.25	8.63	10.67
MEI-Med	12.62	9.94	10.76	9.90	11.21	12.16	10.80	11.06	10.00	10.94
MEI-ML	15.15	13.35	12.41	11.24	10.15	9.43	9.70	9.70	9.07	11.13
Mean	13.21	10.95	10.62	10.84	10.72	10.71	10.27	9.93	9.06	10.70

*Values are given in dB.*

**TABLE 4:** Standard deviations of sound pressure level (SPL) for each categorical unit (CU) for participants with hearing loss for each of the four CLS procedures at 4 kHz.

**CU**	**5**	**10**	**15**	**20**	**25**	**30**	**35**	**40**	**45**	**Mean**
Fixed-level	12.43	11.86	12.59	13.05	13.62	13.47	12.87	10.63	11.68	12.47
Slope-adaptive	14.46	13.80	13.16	14.56	14.03	14.00	12.90	12.40	11.96	13.47
MEI-Med	11.52	12.00	11.08	11.53	13.75	13.02	12.91	11.25	12.80	12.21
MEI-ML	16.83	15.41	15.40	15.53	15.31	14.73	14.23	13.74	12.14	14.81
Mean	13.81	13.27	13.06	13.67	14.18	13.81	13.23	12.00	12.14	13.24

*Values are given in dB.*

**FIGURE 5:**
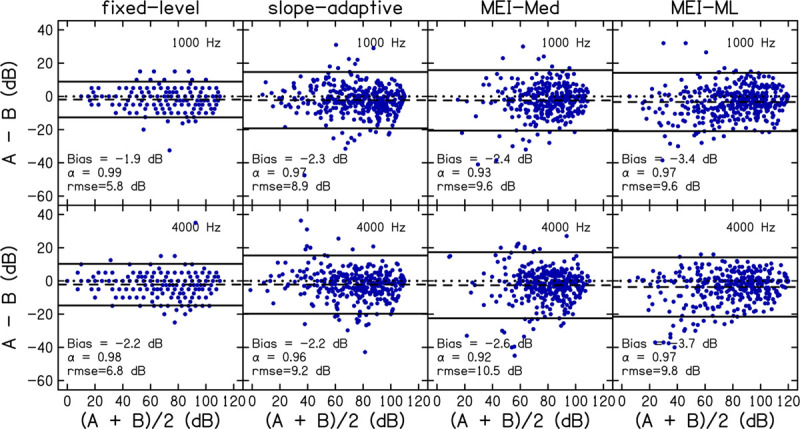
Reliability was assessed by comparing data for visit 1 to visit 2. Panels are Bland-Altman plots for each CLS procedure at 1 (top row) and 4 kHz (bottom row). A = visit 1; B = visit2. Data points represent the difference between visits (A–B; *y*-axis) compared to the mean of both visits [(A + B)/2; *x*-axis]. The dashed line represents the bias. For references, a difference of zero is indicated using a dotted line. The solid lines represent the 95% limits of agreement. Values of Bland-Altman bias, Cronbach’s α, and root-mean-square errors (rmse) are displayed as insets in each panel and in Table 5.

Reliability was assessed by comparing CLS functions from the first visit to those obtained on the second visit. Test–retest reliability for the fixed-level, slope-adaptive, MEI-Med and MEI-ML procedures are displayed in Figure 5. Panels are Bland-Altman plots for each CLS procedure. Values of Bland-Altman bias, Cronbach’s α, and rmse are displayed as insets in each panel and in Table 5. Bland-Altman bias was <|4| and values for Cronbach’s α were ≥0.9 for all procedures, indicating excellent reliability. As expected, the fixed-level was the most reliable CLS procedure because it utilized a larger number of trials compared to the adaptive procedures.

**TABLE 5:** Reliability was assessed by comparing data for each of the four CLS procedures from visit one to visit two.

	**1 kHz**			**4 kHz**		
	**B&A bias**	**α**	**rmse**	**B&A bias**	**α**	**rmse**
Fixed-level	–1.90	0.99	5.81	–2.21	0.98	6.75
Slope-adaptive	–2.29	0.97	8.92	–2.19	0.96	9.20
MEI-Med	–2.43	0.93	9.58	–2.61	0.92	10.46
MEI-ML	–3.38	0.967	9.57	–3.66	0.97	9.81

*Bland-Altman (B&A) bias, Cronbach’s α, and root-mean-square errors (rmse) are reported.*

**FIGURE 6:**
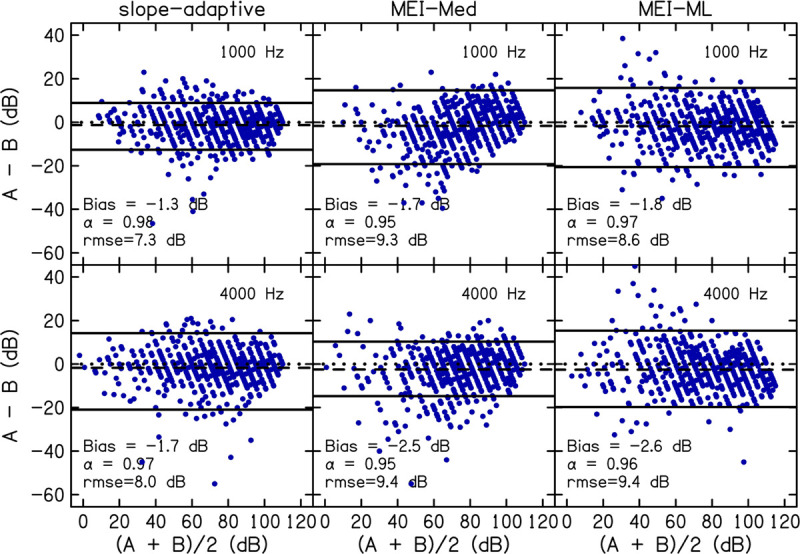
Accuracy was assessed by comparing slope-adaptive and MEI-adaptive procedures to the fixed-level procedure. Panels are Bland-Altman plots for each CLS procedure at 1 (top row) and 4 kHz (bottom row). A = fixed-level procedure; B = adaptive procedure. Data points represent the difference between the fixed-level and adaptive procedure (*y*-axis) compared to the mean of the fixed-level and adaptive procedure (*x*-axis). The dashed line represents the bias. The solid lines represent the 95% limits of agreement. Bland-Altman bias, Cronbach’s α, and root-mean-square errors (rmse) are displayed as insets in each panel and in Table 6.

Accuracy was assessed by comparing the slope-adaptive and MEI-adaptive procedures to the fixed-level procedure. Bland-Altman plots are shown in Figure 6 for each CLS procedure. Values of Bland-Altman bias, Cronbach’s α, and rmse are displayed as insets in each panel and in Table 6. As with the reliability analysis, values for Cronbach’s α were ≥0.9 for all procedures, indicating excellent internal consistency. Bland-Altman bias was <|3|. The accuracy was best for the slope-adaptive procedure. The accuracy was better for MEI-ML than for MEI-Med, but neither is as good as the slope adaptive-procedure.

**TABLE 6:** Accuracy was assessed by comparing the three adaptive procedures to the Fixed-Level procedure across both visits.

	**1 kHz**			**4 kHz**		
	**B&A bias**	**α**	**rmse**	**B&A bias**	**α**	**rmse**
Slope-adaptive	–1.34	0.98	7.28	–1.73	0.97	8.02
MEI-Med	–1.70	0.95	9.31	–2.49	0.95	9.44
MEI-ML	–1.83	0.97	8.64	–2.57	0.96	9.44

*Bland-Altman (B&A) bias, Cronbach’s α, and root-mean-square errors (rmse) are reported.*

**FIGURE 7:**
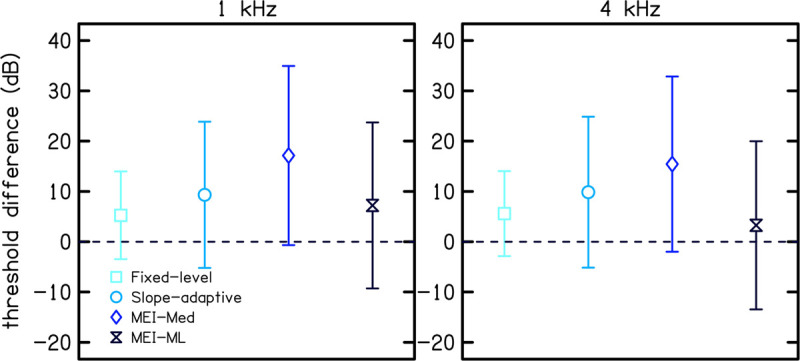
Threshold differences between CLS thresholds and audiometric thresholds for each of the four CLS procedures. The difference in threshold was calculated by subtracting the audiometric threshold from the CLS threshold. Symbols represent the mean difference and error bars represent one standard deviation.

Figure 7 shows the difference between CLS estimates of threshold and audiometric thresholds for each CLS procedure. The difference in thresholds were calculated by subtracting audiometric threshold from the CLS threshold. CLS thresholds were higher than audiometric thresholds for all four procedures (difference >0 in Figure 7). However, error bars included zero for all four CLS procedures.

## Discussion

The evaluation and diagnosis of abnormalities in loudness perception in a variety of patient populations may benefit from improvements in the reliability and accuracy of CLS measurement procedures. Cochlear damage, including sensorineural hearing loss, leads to reduced dynamic range, and in some cases, hyperacusis and/or tinnitus. An attractive feature of adaptive procedures for CLS is that levels that are too uncomfortable that one would not like to listen to again are not presented, allowing for measurement of loudness in listeners who may have hyperacusis. Incorporating individual loudness measures in the hearing aid fitting may improve listener satisfaction and device acceptance. However, CLS measurements have not been accepted by clinicians, partly due to the time required to obtain them. On average, the standard fixed-level CLS procedure took approximately 15 min per frequency. The test time for the MEI-adaptive procedure was, on average, reduced to approximately 3 min per frequency, increasing the feasibility of including loudness measures in clinical practice.

Overall, reliability and accuracy were excellent at both 1 and 4 kHz (Cronbach’s α > 0.9). Both accuracy and reliability were better at 1 kHz than 4 kHz (higher α and lower absolute bias). This perhaps reflects the fact that our listeners had greater hearing loss at 4 kHz than 1 kHz (see Figure 1).

The CLS functions plotted in Figures 3, 4 are averages of measurements collected over two visits. The participant with mild HL represented in Figure 3 did not use CU 40 or 45 in the 1 kHz slope-adaptive procedure on the first visit (though they rated 110 dB SPL as “Too Loud” on visit 2), thus reducing the data for those CUs. This variability is common in human behavioral data. Overall, the trends in the group data (Figure 4) were consistent with those of the individual data (Figure 3).

Across procedures, SDs were higher for lower CUs compared to higher CUs, similar to trends noted in Trevino et al. (2016a). The variability was similar between participants with NH and HL at 1 kHz but was increased for participants with hearing loss at 4 kHz. This contrasts with previous studies that observed higher variability for participants with NH than for participants with hearing loss (Brand and Hohmann, 2002; Rasetshwane et al., 2015). Larger variability of CLS functions for participants with NH compared to participants with sensorineural HL is expected because participants with NH have a wider dynamic range and thus a wider range of possible SPLs that they can assign to a particular loudness category. The observed discrepancy remains unexplained.

In the Bland-Altman analysis of reliability and accuracy (Figures 5, 6), the distribution of differences between two sets of measurements (A−B) is plotted against the mean of the measurements [(A + B)/2]. Measurement bias, which represents systematic error, is calculated as the mean of the differences, and should be close to zero for repeatable measurements. Whether the bias is negative or positive is not important. A bias <0 simply means that measurement B is larger in magnitude/amplitude compared to measurement A.

Interpretation of the LOA for the Bland-Altman plot requires prior information regarding what is considered a significant change in the measurement being analyzed. As an example, hearing conservation programs consider a change in audiometric threshold of 10 dB as a significant threshold shift. Thus, a Bland-Altman analysis for accuracy or repeatability of audiometric threshold can utilize 10 dB to interpret the LOA. Unfortunately, prior work has not defined a significant change in CLS data that can be applied to interpret Bland-Altman analyses. Therefore, the analyses were complemented with Cronbach’s alpha. An attractive feature of Cronbach’s alpha is that there are published guidelines for interpreting the outcome, and the interpretation is not dependent on the type of measurement.

Figure 7 compares CLS estimates of hearing thresholds (i.e., the stimulus level corresponding to 2.5 CU obtained by extrapolation using linear regression) to audiometric thresholds for each CLS procedure. In particular, Figure 7 shows the mean difference between CLS and audiometric thresholds across participants. Threshold differences were greater than zero for all four CLS procedures, indicating that CLS estimates of thresholds were higher than audiometric thresholds. Of the adaptive procedures, the MEI-ML method resulted in the best estimate of thresholds (difference = 7 and 3 dB at 1 and 4 kHz, respectively). This is likely due to the smoothing across loudness categories that was done for this procedure. Our observation of higher thresholds for CLS compared to audiometric testing is in contrast with that of Trevino et al. who reported that, on average, CLS thresholds were lower than audiometric thresholds, with differences up to 20 dB. The discrepancy between the two studies may be due to the differences in the study populations.

Consistent with Moore (2004), our procedure for estimating CLS threshold is not thought to be related to the concept of softness imperception (abnormally large loudness at absolute threshold; Buus and Florentine, 2002). Unlike other procedures for measuring loudness, CLS relates more to a listener’s experience and informal descriptions of their loudness percepts and includes loudness descriptors such as “Very Soft” and “Soft.” Thus, because listeners did perceive “Soft” sounds in CLS, the concept of softness imperception is not applicable to CLS.

In order to better understand the poorer performance of the MEI procedure, model simulations were conducted by using the same empirical distributions that were used to construct the MCPF catalog. Hundreds of simulated listeners were selected from these empirical distributions and simulated responses in each simulated trial were generated according to the expected performance of the simulated listener. Estimation of simulated CLS functions has multiple advantages. The most common use for probabilistic listener models is to support the development of experiments or listening devices. They also allow for the application of concepts from detection, information, and estimation theory to the analysis of results and methodology of the experiment (Trevino et al., 2016b). The simulations were implemented using Monte Carlo methods, and therefore accounted for the randomness of individual listeners.

**FIGURE 8:**
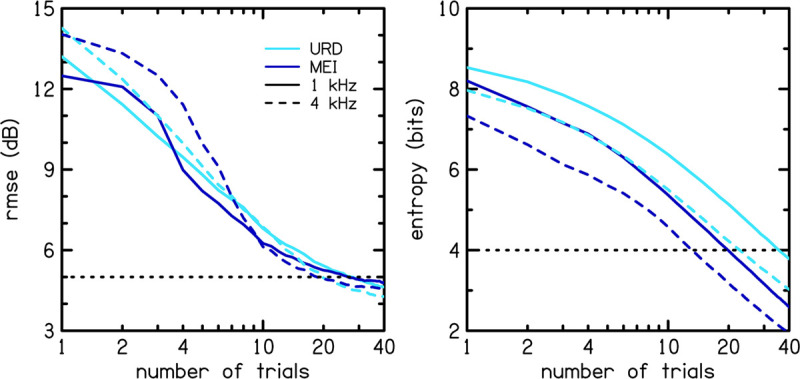
Model simulation from a catalog created in previous work (Trevino et al., 2016a). Root-mean-square error (rmse; left panel) and entropy (right panel) for the MEI algorithm (dark blue lines) are compared to those for the Uniform Random Distribution (URD; light blue lines) procedure. The solid lines represent 1 kHz and the dashed lines represent 4 kHz.

**FIGURE 9:**
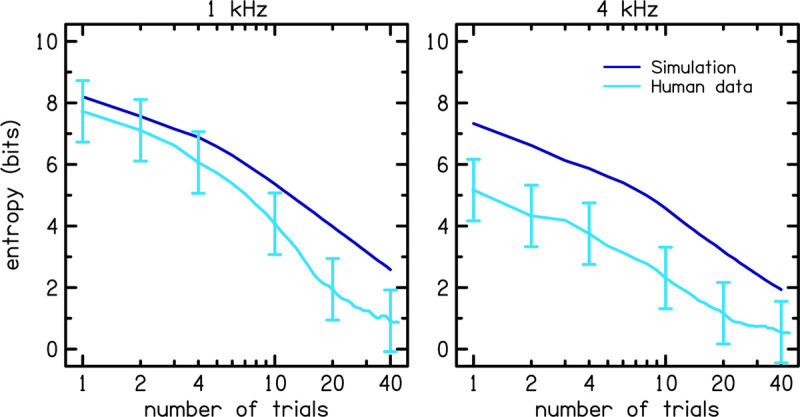
Comparison of the entropy between the simulation (dark blue) and human data (light blue). Error bars for the human data represent one standard deviation from the mean.

Figure 8 shows rmse (left panel) and entropy (right panel) for the MEI algorithm (dark blue lines) compared to those for the Uniform Random Distribution (URD; light blue lines) procedure. URD was used to simulate the adaptive ISO procedure by randomly selecting the next stimulus level from a uniform distribution, or range of possible levels that each have equal probability. The solid lines represent 1 kHz and the dashed lines represent 4 kHz. The fact that MEI consistently outperforms URD in terms of entropy reduction (right panel) tells us that the tracking implementation is performing as well as expected. However, the fact that MEI is not consistently better than URD in terms of rmse reduction tells us that the MCPF catalog lacks sufficient information to improve the accuracy of the adaptive tracking procedure. Comparison of the simulated entropy reduction with the human data in Figure 9 further validates the MEI implementation by showing greater entropy reduction in the human listeners (light blue) compared to the simulation (dark blue) for 1 (left panel) and 4 kHz (right panel). Error bars for the human data represent one SD from the mean. Entropy is lower for the human data compared to the simulation. This result rules out the possibility that the observed poorer performance was due to flawed implementation of the MEI tracking methods, which implicates intrinsic uncertainty in the MCPF catalog as the factor that currently limits MEI performance.

In summary, the MEI tracking apparently produced less accurate CLS functions compared to the other tracking methods because of inherent uncertainty in the MCPF which reflects the uncertainty of the listeners on whom the MCPF catalog was based, and not because the MEI procedure was improperly implemented or lacked the ability to reduce catalog entropy. The results of this study indicate that our measure of entropy was not sufficiently correlated to rmse to produce more reliable CLS functions.

Further investigation is warranted to understand how the MCPF catalog could be modified to achieve closer correspondence with catalog entropy. The MCPF catalog would be improved by reconstructing it from new fixed-level CLS data at a larger number of stimulus frequencies and a more uniform representation of hearing-loss categories. However, such an improved MCPF catalog would not necessarily improve MEI efficiency. Relaxing the restriction on large level transitions (45 dB for the current study), which can bias listener responses, or by including catch trials where listener biases are expected, may improve performance of the MEI-adaptive method. During a CLS test, large transitions in SPL as well as presentations of multiple consecutive trials at similar SPLs are avoided as these can bias listener responses. For example, if a presentation of 10 dB SPL is followed by a presentation of 80 dB SPL, listeners will perceive the 80 dB SPL signal as louder than if it followed a 50 dB SPL signal. Thus, changes were made to our MEI-adaptive approach to accommodate listener effects that can bias CLS data. Unfortunately, these changes, although necessary for practical purposes, resulted in a suboptimal MEI-adaptive procedure. Thus, there is potential to improve the performance of the MEI-adaptive procedure. Such modifications could improve both MEI tracking efficiency and ML estimation of CLS functions. Further improvements in the reliability and accuracy of CLS could enhance the clinical acceptability of loudness measurements and potentially improve hearing aid fitting methods.

## Data Availability Statement

All datasets and analysis code generated for this study are located online at OSF (Open Science Framework) https://osf.io/xwb6p/.

## Ethics Statement

The studies involving human participants were reviewed and approved by Institutional Review Board, Boys Town National Research Hospital. The patients/participants provided their written informed consent to participate in this study.

## Author Contributions

SF collected the data and wrote the manuscript. JK contributed to experiment design. SN and DR designed the study, reviewed the data, and provided interpretive analysis and critical revisions. All authors discussed the results and implications and commented on the manuscript at all stages.

## Conflict of Interest

The authors declare that the research was conducted in the absence of any commercial or financial relationships that could be construed as a potential conflict of interest.
